# Sidération myocardique au cours d'une intoxication au monoxyde de carbone (CO) chez une femme enceinte

**DOI:** 10.11604/pamj.2015.21.66.6081

**Published:** 2015-05-28

**Authors:** Mahamadoun Coulibaly, Mohamed Adnane Berdai, Smael Labib, Mustapha Harandou

**Affiliations:** 1 Service d'Anesthésie-Réanimation Mère-enfant, CHU Hassan II de Fès, Fès, Maroc

**Keywords:** Monoxyde de carbone (CO), grossesse, sidération myocardique, troponine, oxygénothérapie hyperbare, carbon monoxide, pregnancy, myocardial stunning, troponine, hyperbaric oxygen therapy

## Abstract

L'intoxication au monoxyde de carbone (CO) est la première cause de décès par intoxication en France. La littérature est ancienne et peu connue. Les signes les plus fréquents de l'intoxication sont la triade: Céphalées; asthénie, faiblesse musculaire surtout des membres inférieurs. Ses conséquences sont potentiellement graves pour le fœtus quand elle survient chez la femme enceinte, il est particulièrement exposé au risque d'hypoxie en raison de la forte affinité de son hémoglobine pour le CO qui traverse aisément le placenta. Les événements cardiovasculaires ne sont pas rares et peuvent être responsable d'une morbi-mortalité assez importante qui peuvent être d'apparition rapide ou secondaire mais régressent habituellement en quelques jours. Des SCA peuvent survenir lors d'une une intoxication au CO avec à l'extrême infarctus myocardique avec surélévation du segment ST. Il paraît légitime de proposer pour toutes les patientes: l’éloignement maternel de la source de CO; l'oxygénothérapie à 100% au masque facial par les services de secours et pendant le transfert; le traitement par oxygénothérapie hyperbare pour toutes les femmes enceintes, le plus rapidement possible et quelque soit l’âge gestationnel.

## Introduction

L'intoxication au monoxyde de carbone (CO), gaz incolore et inodore, est le plus souvent accidentelle et met en cause un appareil de chauffage au gaz défectueux, l'obstruction d'un conduit de cheminée, l’émission de gaz d’échappement d'un véhicule en milieu confiné ou de fumées lors d'un incendie [1], elle est la première cause de décès par intoxication en France et dans les pays industrialisés. Ses conséquences sont potentiellement graves pour le fœtus quand elle survient chez la femme enceinte, il est particulièrement exposé au risque d'hypoxie en raison de la forte affinité de son hémoglobine pour le CO qui traverse aisément le placenta. Les symptomatologies neuropsychologiques sont les plus fréquents en cas d'inhalation de CO [2], cependant les événements cardiovasculaires ne sont pas rares et peuvent être responsable d'une morbi-mortalité assez importante qui peuvent être d'apparition rapide ou secondaire mais régressent habituellement en quelques jours. Nous rapportons l'observation d'une Patiente en cours de grossesse ayant été accidentellement intoxiquée au CO.

## Patient et observation

Il s'agit d'une patiente de 18 ans, non fumeuse primigeste à 16 SA. Admise aux urgences après avoir été découverte inconsciente dans la douche (environ 1 heure d'exposition d'après la famille); l'utilisation de chauffe eau non conforme a fait évoquer le diagnostic d'intoxication au monoxyde de carbone. A son admission à l'hôpital, la patiente était somnolente GCS: 13, apathique, se plaignant de douleurs thoracique, tachycarde à 120 battements par minutes, polypnéique à 25 cycles par minutes, les conjonctives étaient normo colorées; l'examen gynéco-obstétrique était normal notamment RCF normal réactif et absence d'autres signes faisant évoquer une souffrance fœtale ou d'avortement, le reste de l'examen clinique était sans particularité. Apres mis en condition et monitorage: spo2:85%; GDS: PH:7, 43, PCO2:29, Po2:193, Hco3-:18,7, Sao2:99%; le bilan biologique a révélé: HBCO:11%; Troponine 3,34 ng/ml (VN <0,08ng/ml) CPK: 310 UI/l; mis à part une légère cytolyse hépatique, le reste du bilan biologique était normal. L’électrocardiogramme trouvait une tachycardie sinusale sans trouble de conduction ou de la répolarisation, de même que l'echodoppler cardiaque qui était sans particularité avec une fonction systolique normale et une fraction d’éjection FE à 65%. La patiente a été mise sous oxygénothérapie au masque haute concentration à 12l/min dès son admission (non disponibilité de l'oxygénothérapie hyperbare OHB). L'etat de conscience était normale après 1h d'oxygénothérapie avec bonne orientation dans le temps et l'espace sans notion d'amnésie antérograde ou autre signe neuropsychique. La patiente fût déclaré sortante de la réanimation âpres 72h d'hospitalisation et âpres normalisation des paramètres biologique (troponine); le suivi de la grossesse a été rigoureuse et a été de déroulement normale; la patiente a accouché d'un nouveau né masculin de 3500 grammes à 39 SA, Apgar 10/10; avec un développement psychomoteur absolument normal. Il est actuellement âgé de 4 mois.

## Discussion

L'intoxication au monoxyde de carbone (CO) est la première cause de décès par intoxication en France. La littérature est ancienne et peu connue. Les signes les plus fréquents de l'intoxication sont la triade: Céphalées; asthénie, faiblesse musculaire surtout des membres inférieurs [3]; cependant ces signes restent non spécifique et l'absence de symptômes est aussi possible lors d'intoxications modérés [4]; dyspnée et polypnée peuvent être observés. L'intoxication au CO est avérée si le taux d'HbCO dépasse 6% chez les non-fumeurs et 10% chez les fumeurs et ce taux est très peu corrélé à la sévérité de l'issue. En effet, ce taux peut être très inférieur à celui du pic réel d'HbCO, notamment si le temps écoulé depuis l'exposition est important ou si de l'oxygène a été administré lors du transport. Il existe trois mécanismes physiopathologiques de la toxicité du CO [5]. Le CO entraîne une diminution du transport de l'oxygène(O_2_), Il se fixe sur l'hémoglobine avec une affinité plus importante que l'O_2_, formant ainsi l'HbCO. L'HbCO induit de plus un déplacement vers la gauche de la courbe de dissociation de l'oxyhémoglobine, diminuant le rélargage de l'O_2_ dans les tissus. Le CO se fixe également aux protéines telles que la myoglobine et le cytochrome a3 et perturbe l'utilisation de l'O_2_ au niveau tissulaire, notamment par le myocarde. Enfin, il existe une toxicité cellulaire directe. Le fœtus est particulièrement vulnérable au CO, qui est souvent tératogène, le passage du CO de la mère vers le fœtus se fait de manière passive facilité également par le cytochrome P450 [6], ce passage est d'autant plus important que le gradient de pression en HBCO est élevé entre la mère et le fœtus, il augmente avec l’âge gestationnel, le poids fœtal ainsi qu'avec l'augmentation du flux sanguin placentaire et de la concentration d'hémoglobine maternelle [7, 8]. La sévérité de l'atteinte fœtale est très variable, allant de l'absence totale d'atteinte à la survenu d'une mort in utero. Quel que soit l’âge gestationnel, l'organe le plus touché reste le cerveau fœtal. Les lésions du tronc cérébral, du cervelet et de la moelle sont rares accompagnant seulement les lésions hémisphériques massives. Les troubles neurologiques sont variables et non spécifiques et se traduisent par une hypotonie, une aréflexie, des crises convulsives, un retard moteur ou mental, ou une microcéphalie. Les signes cardio-vasculaires: œdème aigu pulmonaire, collapsus, troubles du rythme et insuffisance coronaire concernent plus d'un tiers des patients avec une intoxication modérée à sévère [9]. L'exposition au CO favorise les évènements thrombotiques aigus par liaison au fibrinogène [10], par augmentation de l'agrégabilité plaquettaire et polyglobulie, et favorise le vasospasme coronaire [11]. Des SCA peuvent survenir lors d'une une intoxication au CO avec à l'extrême infarctus myocardique avec surélévation du segment ST mais aussi en l'absence d'anomalie coronaire, réalisant un tableau de sidération myocardique [12] comme chez notre patiente. Le sujet indemne de toute pathologie cardiaque peut développer un véritable état de choc cardiogénique par ischémie diffuse (sidération myocardique) au cours d'une intoxication au CO. Les séquelles cardiaques au décours d'une intoxication au CO peuvent être seulement électrocardiographiques (persistance de troubles de la repolarisation) ou cliniques, avec la persistance de signes d'insuffisance cardiaque, dont le degré dépend de l'importance de l'ischémie myocardique initiale et du terrain sous-jacent. La fréquence de ces séquelles n'a pas été évaluée.

La prise en charge comprend éviction de la source et oxygénothérapie. Reconnue depuis 1895 [13], elle peut être normobare ou hyperbare et permet la dissociation de la carboxyhémoglobine. La dissociation des autres complexes est plus lente et ne commence que lorsque la délivrance périphérique de l'O_2_ est satisfaisante. L'oxygénothérapie normobare au masque à haute concentration doit être débutée le plus tôt possible à haut débit. L'OHB est recommandée en cas de trouble de conscience, d'anomalie objective à l'examen neurologique, de perte de connaissance initiale et chez la femme enceinte [14], l'oxygénothérapie hyper-bare est associée à des atteintes fœtales en moyenne moins sévères. Elle accélère la dissociation du CO de l'hémoglobine et des protéines de la chaîne respiratoire mitochondriale; raccourcit la demi-vie de l'HbCO, augmente la concentration de l'oxygène dans le sang et diminue la production de radicaux libres [7]. On estime que la demi-vie de l'HbCO maternelle est de quatre heures en air ambiant, de 1h30 en oxygénothérapie normobare et seulement de 20 minutes en oxygénothérapie hyperbare à 3 ATA. L'atteinte de l’état d’équilibre et le temps d’élimination du CO étant plus long chez le fœtus que chez sa mère, un traitement en urgence peut théoriquement prévenir la constitution de lésions fœtales, diminuer son œdème cérébral et ses séquelles neurologiques à long terme [15]. Du fait de cette élimination plus lente du CO fœtal, la durée de l'oxygénothérapie hyperbare devrait théoriquement être augmentée chez la femme enceinte [16]. Des auteurs [16] ont proposé de retenir comme indication: un taux maternel d'HbCO supérieur à 20%, la présence de signes cliniques d'intoxication au CO, l'existence d'anomalies du rythme cardiaque fœtal. Ces auteurs préconisent de poursuivre cette oxygénothérapie hyperbare si les signes maternels ou fœtaux persistent 12 heures après le début de la première séance [16, 17]. En France, les recommandations concernant la conduite à tenir en cas d'intoxication oxycarbonée datent de 2005 [18]. Il paraît légitime de proposer pour toutes les patientes: l’éloignement maternel de la source de CO; l'oxygénothérapie à 100% au masque facial par les services de secours et pendant le transfert; le traitement par oxygénothérapie hyperbare pour toutes les femmes enceintes, le plus rapidement possible et quelque soit l’âge gestationnel. Il est indéniable que l'oxygénothérapie hyperbare améliore le pronostic maternel et fœtal, cependant plusieurs paramètres interviennent dans ce pronostic comme nous le voyons dans le Tableau 1: l’âge gestationnel, la durée de l'exposition, le taux d'HBCO à l'admission, la rapidité de l'oxygénothérapie normo ou hyperbare sont entre autre des facteurs pronostique de l'intoxication au monoxyde de carbone chez la femme enceinte.

**Tableau 1:** Synthèse de quelques cas de la littérature d'après [6]

Auteur	Année	Nombre de cas	Enfants atteints	Age gestationnel à l'exposition	Taux d'HbCO maternel (%)	Oxygéno-thé rapie hyperbare	Issue
Margulies	1986	1	0	37	59	Non	Normale
Hollander et al.	1987	1	0	38	6	oui	Normale
Caravati et al.	1988	6	3	20	9	non	normale
Abboud et al.	2001	2	1	34	18	oui	Lésions cérébrales
Alehan et al.	2007	1	1	20	-	Non	Atteinte noyaux gris
Towers et Corcoran	2009	3	0	32	36	oui	Normale
Yildiz et al.	2010	1	1	33	25	Non	Encéphalopathie

## Conclusion

L'intoxication au monoxyde de carbone est la première cause de décès par Intoxication accidentelle dans le monde. Elle est associée à une mortalité et une morbidité importantes. La seule notion de l'exposition au risque doit faire évoquer le diagnostic d'intoxication au monoxyde de carbone en situation d'urgence. Les complications, essentiellement neurologiques et cardiorespiratoires, peuvent engager le pronostic vital à court terme, et requièrent un traitement urgent, dont l'oxygène est la pierre angulaire. L'intoxication au CO pendant la grossesse peut mettre en jeu le pronostic vital de la mère dans les formes sévères et avoir des conséquences majeures sur le fœtus et son cerveau même dans les formes maternelles modérées. La prévention est un élément indispensable de la prise en charge globale de ce type d'intoxication. Elle relève du contrôle régulier des sources potentielles, de l'installation généralisée de détecteurs.
